# Polyvinyl Alcohol/Chitosan/Polyhexamethylene Biguanide Phase Separation System: A Potential Topical Antibacterial Formulation with Enhanced Antimicrobial Effect

**DOI:** 10.3390/molecules25061334

**Published:** 2020-03-15

**Authors:** Yunzhou Ni, Zhixiang Qian, Yu Yin, Weien Yuan, Fei Wu, Tuo Jin

**Affiliations:** School of Pharmacy, Shanghai Jiao Tong University, Shanghai 200240, China; 820876094@sjtu.edu.cn (Z.Q.); yuanweien@126.com (W.Y.); feiwu@sjtu.edu.cn (F.W.)

**Keywords:** polyvinyl alcohol, chitosan, polyhexamethylene biguanide, phase separation, enhanced antimicrobial effect, low dose, topical antibacterial formulation

## Abstract

An aqueous polyvinyl alcohol (PVA)/chitosan (CHT)/polyhexamethylene biguanide (PHMB) blends (PVA/CHT/PHMB blends) has been developed as a potential low dose topical antibacterial formulation with enhanced antimicrobial effect. The preparation of PVA/CHT/PHMB blends was quite facilely, with just dissolved PVA, CHT, PHMB in water in order. There was the aggregates with 100 nm size around induced by phase separation in the blends and an aqueous two-phase system (ATPS) formed, as non-ionic polymer PVA formed a continuous phase and cationic polymer CHT and PHMB formed dispersed phases. The minimum inhibitory concentration (MIC) of PHMB in the PVA/CHT/PHMB blends was 0.5μg/mL, which was four times lower than the MIC of PHMB individually. A phase separation increased zeta potential mechanism was proposed to explain the enhanced antibacterial activities. In addition, the blends could easily form film on the skin surface with good water vapor permeability and be used as a liquid bandage to accelerate the scratch wound healing process of nude mouse. These findings provide experimental evidence that the PHMB-functionalized blends could be further explored as low-dose topical antibacterial formulations, and the nano-sized phase separation strategy could be used to design novel low-dose topical antibacterial formulations with an enhanced antimicrobial effect.

## 1. Introduction

Topical antibacterial agents [1,2] are widely used to treat localized infections, and also can be used as prophylactic agents to prevent infection following dermatologic surgery, abrasions, cosmetic procedures. Although adverse effects of topical antibacterial agents are rare, allergic contact dermatitis is well documented [3]. Generally, side effects are dose responsive, as the incidence of side effects are fewer with low dose antibacterial therapy [4,5]. Hence, the development of novel low dose antibacterial system with effective antibacterial effects is important and necessary. Combination drugs yielding synergistic effect is an option for antibacterial therapy [6], which can reduce the dose of antibacterial agents. Therefore, it is proposed to develop a new combination strategy which could enhance antibacterial activity with low drug dose.

In recent years, nanotechnology [7] has been introduced into the antibacterial research area and achieved significant progress. Kinds of nanocarriers including micelles [8], liposomes [9], dendrimers [10], and nanogels [11] etc., have been developed. Generally, the fabrication process requires the preparation of nanocarriers first, and then introduce an antibacterial agent into nanocarriers via kinds of methods. However, these methods usually involve several preparation steps, and the high yield of antibacterial nanocarriers is a challenge. Therefore, it is worth developing novel facile strategies to prepare antibacterial agent containing nanocarrier systems. 

Polyhexamethylene biguanide (PHMB) [12,13] is one kind of cationic polymer and has broad-spectrum antibacterial activities with high therapeutic index. It has attracted wide attention as antimicrobial agent against many pathogens [14] including Gram-positive bacteria, Gram-negative bacteria, fungi and certain viruses. PHMB is relatively safe when it is used on skin, eyes, and epithelium of wounds. It can be available as disinfecting solution [15] and also can be impregnated into dressings [16]. Some recent studies reported that nanocarriers had been used to delivery PHMB to obtain better antibacterial effects. Elnaz et al. [17] prepared PHMB loaded nano cationic liposome with a smaller size (34 nm) and found its antibacterial activity was enhanced against *Staphylococcus aureus* (*S. aureus*) and *Escherichia coli* (*E. coli*). Sumaira et al. [18] developed PHMB-functionalized silver nanoparticles and found that its antibacterial activity was about 100 times higher than previous report. However, reports on facilely preparation PHMB-functionalized nano-systems were still few.

**Scheme 1:**
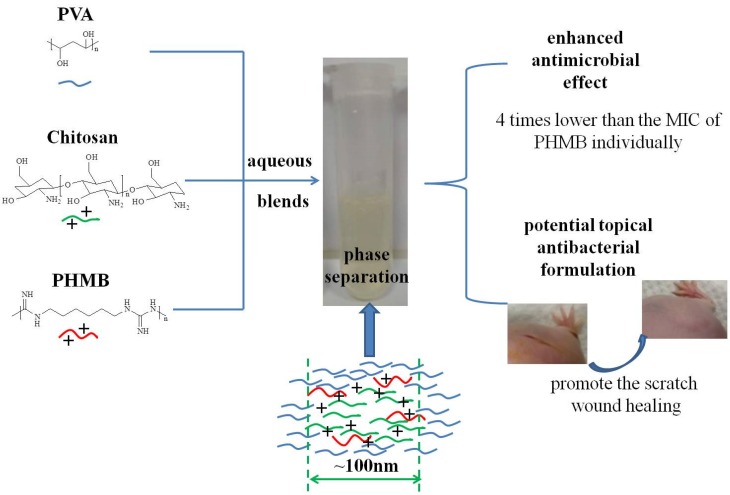
Schematic illustration of preparation of polyvinyl alcohol/chitosan/polyhexamethylene biguanide blends (PVA/CHT/PHMB blends) as a potential topical antibacterial formulation with enhanced antimicrobial effect.

The objective of this study was to develop a novel facile strategy to prepare a low dose PHMB-functionalized nano-system as a potential topical antibacterial formulation with enhanced antimicrobial effect. As illustrated in Scheme 1, the fabrication process of PVA/CHT/PHMB blends was just dissolved PVA, CHT, and PHMB in water in order, which was highly simple. The low dose PHMB-functionalized blends exhibited the enhanced antibacterial effect and the phase separation between PVA and CHT and PHMB was supposed to be responsible for it. Interestingly, the blends could promote scratch wound healing of nude mouse. These aspects make this method attractive from a topical antibacterial formulation point of view.

## 2. Materials and Methods 

### 2.1. Materials

Chitosan Hydrochloride (DAC degree 80.0–90.0%) was as a gift from Zhejiang Golden-Shell Pharmaceutical Co. Ltd. Poly(vinyl alcohol) (M_W_ 8000~10,000, 87–89% hydrolyzed) were purchased from Sigma-Aldrich (Shanghai, China). Poly(hexamethylenebiguanide) hydrochloride were purchased from Macklin Inc (Shanghai, China). The water used in all experiments was prepared using a Milli-Q purification system (Millipore Corporation, Bedford, MA, USA) and had a resistivity greater than 18.2 MΩ∙cm. All other chemicals were purchased from domestic suppliers and used as received. Clear polystyrene tissue culture treated 96-well plates were obtained from Corning Costar (Corning, New York, NY, USA). 

### 2.2. Preparation of PVA/CHT/PHMB Blends

PVA, CHT and PHMB were dissolved in water in order at room temperature (25 °C) to obtain the PVA/CHT/PHMB blends. Typically, 1 g PVA was firstly put into 20 mL water with stirring for 20 min, and PVA (5%) solution was obtained. Then, 600 mg CHT was put into 20 mL PVA (5%) solution with stirring for 6 h, and PVA (5%)/CHT (3%) blends was obtained. 10 μg PHMB was put into 20 mL PVA (5%)/CHT (3%) solution with stirring for 10 min, and PVA (5%)/CHT (3%)/PHMB (0.5 μg/mL) blends was obtained. The preparation process of the blends was carried out at ambient condition.

### 2.3. Bacterial Strains and Culture Conditions

Two well characterized *Staphylococcus aureus* and *Escherichia coli* strains were obtained from the American Type Culture Collection. The bacteria were cultured in cation-adjusted Mueller–Hinton broth at 35 ± 2 °C. Prior to incubation with samples, the bacteria were cultured overnight in 5 mL of Mueller Hinton broth in a incubation shaker at 37 °C, 150 rpm until the culture reached OD_600_ of 1.0, corresponding to 10^9^ CFU/mL. The overnight cultures were diluted to 10^7^ CFU/mL with sterile broth.

### 2.4. Assays for Antibacterial Activity

The minimum inhibitory concentration (MIC) was determined by a microdilution method. The samples for antibacterial activity were prepared as follows: for PVA/CHT/PHMB blends, polyvinyl alcohol (PVA, 5%), CHT (3%) and PHMB (1024 μg/mL) were in order dissolved in water at room temperature with mild stirring. The plates used in the microdilution experiments were prepared by mixing 100 μL bacterial Mueller–Hinton broth with 100 μL sample solution. The final concentrations of tested antibacterial agents (PHMB) were: 0.25, 0.5, 1, 2, 4, 8, 16, 32, 64, 128, 256 and 512 μg/mL for samples prepared at 37 °C. For PVA/PHMB blends, CHT/PHMB blends, and PHMB, the sample prepared procedures were the same as the procedure of PVA/CHT/PHMB blends. Samples (200 μL each) were dispersed into the wells of the microdilution plates. The inoculated microdilution plates were covered with a plastic lid and incubated at 37 °C for 24 h. Bacterial growth was evaluated visually and the MIC was recorded as the lowest concentration that completely inhibits growth. The number of replicates for MIC assay was 3.

### 2.5. DLS and Zeta Potential Measurement of PVA/CHT/PHMB Blends

DLS and zeta potential measurements were performed on aqueous solutions with a Malvern Zetasizer Nano S (Malvern Instruments, Ltd, Westborough, MA, USA) equipped with a 4 mW He-Ne laser light operating at 633 nm. All samples were measured at a scattering angle of 90°. The number of replicates for DLS and zeta potential measurements was 3.

### 2.6. Rheological Measurement of PVA/CHT/PHMB Blends 

Dynamic, steady-state shear measurements were carried out on a controlled stress rotational rheometer (AR-G2, TA Instruments, New Castle, DE, USA) equipped with a 2°1′8′′ cone plate geometry (60 mm diameter, 58 μm gap). The rheological measurements were conducted at 37 °C. The number of replicates for rheological measurements was 3.

### 2.7. Preparation of PVA/CHT/PHMB Blends Formed Film

Typically, the as-prepared 200 μL PVA/CHT/PHMB blends was cast onto a clean glass plate with a rectangular shape with 10 mm × 20 mm, followed by drying at 30 °C for 6 h. Then, the formed film was peeled off from the glass plate carefully.

### 2.8. Observation of the Surface Morphology of PVA/CHT/PHMB Blends Formed Film

The surface morphology of PVA/CHT/PHMB blends formed film was observed under a Field Emission Scanning Electron Microscope (FEI Co., Hillsboro, OR, USA) operated at 5 kV. The samples were cut into small pieces (5 × 5 mm), and coated with gold using a sputter coater under 15 mA for 60 s.

### 2.9. WVTR (Water Vapor Transmission Rate) of PVA/CHT/PHMB Blends Formed Film

To determine the moisture permeability of PVA/CHT/PHMB blends formed film, WVTR was measured according to ASTM Standard (2010). Briefly, a circular piece of the specimen was mounted on the top of a plastic tube of 28 mm in diameter with 8 mL of distilled water. The tube was then placed into an incubator kept at 30 °C and 40 ± 5% relative humidity (RH). The WVTR was calculated by dividing the daily weight loss of water by the area of the vial opening. 

### 2.10. Mechanical Properties of PVA/CHT/PHMB Blends Formed Film

A universal mechanical test machine (Instron, BAB-20MT, Trans cell Technology Inc., Shanghai, China) was used to determine the mechanical property at room temperature with a stretch velocity of 10%/min. Briefly, samples were cut into a rectangular shape with 10 × 10 × 0.077 mm. The thickness of blends formed film was measured using a micrometer with 0.001 mm accuracy, and the thickness was measured at five different places for each sample.

### 2.11. Scratch Wound Healing Test

Two nude mice were used for the treatment of scratch wounds. They were allocated into two groups, one was treated with PVA/CHT/PHMB blends, another one was no treated. Each one was evaluated at 6 days. All animals were housed in a stable temperature and night/day cycle atmosphere with specific pathogen-free requirement, where the animal surgery was made. We put several cotton balls dipped in diethyl ether on the bottom of a 500 mL glass specimen bottle and close the bottle cap to make the bottle full of diethyl ether vapor. Then, we put the nude mouse into the bottle for anesthesia for about 10~20 seconds, a 10 mm-long skin and 1 mm deep incision was made with a scalpel. One was treated with PVA/CHT/PHMB blends on the wounds, and another one was no treated. During the surgery, the nude mice were kept anesthetized to avoid them to touch the injury. The nude mice were raised in separate cages after surgery. Observations and procedures were performed at 1, 2, 3, 4, 5 and 6 days respectively. Animal care and use were in accordance with the guidelines of the Animal Ethics Committee for Shanghai Jiao Tong University. It has been approved by experimental animal management committee, academic committee and ethics and use committee of Shanghai Jiaotong University (Approval No. A2017073).

## 3. Results and Discussion

### 3.1. The Preparation of Blends

**Figure 1:**
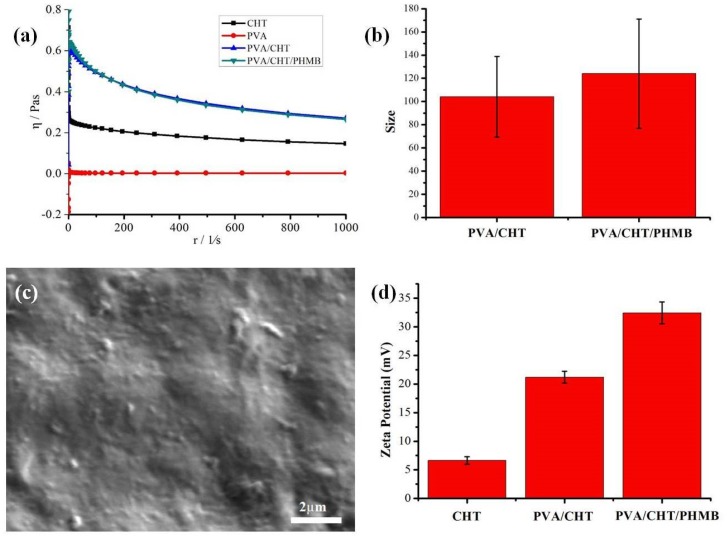
(**a**) The viscosity versus the shearing rate for PVA, CHT, PVA/CHT, PVA/CHT/PHMB solution at 37 °C, (**b**) The size of PVA/CHT and PVA/CHT/PHMB respectively, (**c**) The SEM images of PHMB in PVA/CHT, and (**d**) The zeta potential of CHT, PVA/CHT and PVA/CHT/PHMB respectively.

The blends were obtained via dissolving PVA, CHT and PHMB in water in order at room temperature with mild stirring. Herein, we studied the physical chemical properties of the blends. Usually, viscosity of the fluids was a function of the deformability and packing of the micro-particles or aggregates [19]. The viscosity would decrease with the increasing of shearing rate because the aggregates were disintegrated gradually. Figure 1a showed the curves (obtained at 37 °C) of viscosity (η) versus shearing rate (r) for PVA, CHT, PVA/CHT, PVA/CHT/PHMB blends. The viscosity of PVA/CHT/PHMB and PVA/CHT blends was significantly higher than PVA and CHT solution, and showed shear thinning behavior with the increase of shearing rate. Thus, the shear thinning behavior indicated that the aggregates had formed in the blends and could be disintegrated. In addition, the dynamic laser light scattering (DLS) data (Figure 1b) showed the aggregation with 100 ± 30 nm size in PVA/CHT blends and 120 ± 40 nm size in PVA/CHT/PHMB blends respectively, which further indicated that the phase separation occurred in the blends. Furthermore, the SEM image (Figure 1c) showed 130 ± 50 nm size nano-phase by statistical calculation of 50 aggregations from Figure 1c in PVA/CHT/PHMB blends, which solid disclosed that the phase separation had occurred. 

It was reported that polymer–polymer system could form large aggregates and separate between two different phases because of steric exclusion [20]. In PVA/CHT, PVA/CHT/PHMB blends, CHT and PHMB were hydrochloride salt, which could absorb large amounts of water and induce steric exclusion between non-ionic polymer PVA and cationic polymer CHT and PHMB. Besides steric exclusion, the interaction like H-bonding between CHT and PHMB acted as attractive force to enable CHT and PHMB self-assemble together and formed CHT and PHMB aggregation. Thus, the phase separation occurred in the blends and aqueous two-phase system formed, non-ionic polymer PVA formed continuous phase and cationic polymer CHT and PHMB formed dispersed phase. Obviously, the charge density of CHT and PHMB phase would increase in the ATPS accordingly. As evidence (Figure 1d), the zeta potential of PVA/CHT/PHMB blends (32 ± 3 mV) and PVA/CHT (22 ± 2 mV) blends were higher than those of CHT (7 ± 1 mV) solution. In addition, because the phase separation occurs spontaneously, the fabrication process of the blends would be energy saving and the obtained blends would be stable, which would be beneficial to the long-term preservation. Overall, the above results strongly demonstrated that the 100 nm size around phase separation with increased zeta potential had occurred in the blends.

### 3.2. The Antibacterial Activity of Blends

**Figure 2:**
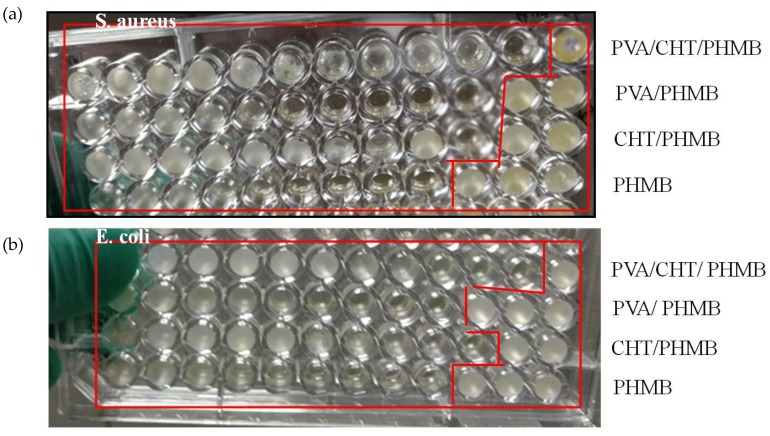
Photograph of minimum inhibitory concentration (MIC) of PHMB, PVA/PHMB, CHT/PHMB, PVA/CHT/PHMB in against (**a**) *Staphylococcus aureus*, and (**b**) *Escherichia coli strains*.

In general, the antibacterial activity of chitosan nanoparticles [21] are higher than that of chitosan due to the higher surface charge density of chitosan nanoparticles. Therefore, the PVA/CHT/PHMB blends with high zeta potential was proposed to exhibit enhanced antibacterial activity. In this study, the order of dissolution was designed to dissolve PVA, CHT, PHMB orderly in order to ensure that PHMB could be assembled on the outer surface of the CHT aggregates, so that PHMB could exert its antibacterial effect. The antibacterial activity of PHMB, PVA/PHMB, CHT/PHMB, PVA/CHT/PHMB blends were compared respectively by MIC assay for evaluating the antibacterial performance [22]. Figure 2 showed the photograph of MIC assay of the samples against *S. aureus* and *E. coli* strains, and Table 1 showed the data of MIC assay. The MIC values of PHMB in the PVA/CHT/PHMB blends was 4 times lower than those of PHMB individually, which clearly indicated that the antibacterial activity of PVA/CHT/PHMB blends was significantly higher than that of PHMB. In addition, the antibacterial activity of PVA/CHT/PHMB blends was better than that of CHT/PHMB because the MIC values of PHMB in the PVA/CHT/PHMB blends was 2 times lower than those of CHT/PHMB. As the MIC values of PHMB in the PVA/CHT/PHMB blends was 0.5 μg/mL only, the blends as a low dose PHMB -functionalized nano-system well exhibited its enhanced antimicrobial effect. 

**Scheme 2:**
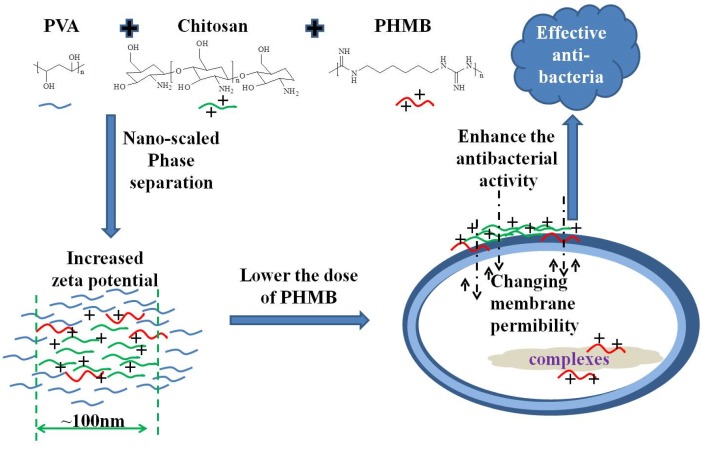
Proposed mechanism of the enhanced antibacterial activity of blends.

Such better antibacterial activity of PVA/CHT/PHMB blends was related with its high zeta potential. The characterization of high zeta potential meant that a number of high positive charge density of CHT/PHMB aggregation was in the blends. Thus, the CHT/PHMB aggregation had stronger affinity with negative charged bacteria cells membrane, and could be tightly adsorbed onto the surface of the bacteria cells, which would disturb the membrane structure and lead to the leakage of intracellular components and efficiently kill the bacteria cells. As the high zeta potential of the PVA/CHT/PHMB blends was originated from the phase separation between PVA and CHT and PHMB. Hence, a phase separation increased zeta potential mechanism (Scheme 2) was proposed to explain the enhanced antibacterial activities of the PVA/CHT/PHMB blends with low dose PHMB. 

### 3.3. PVA/CHT/PHMB Blends Film Forming Characterization

**Figure 3:**
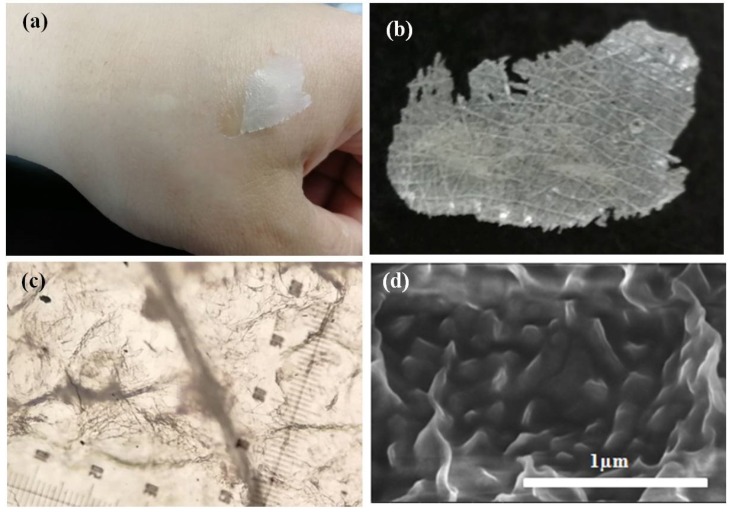
The appearance and properties of L-Bandage formed film. (**a**) Digital photograph of PVA/CHT/PHMB film on the surface of human skin; (**b**) digital photograph, (**c**) optical photograph, (**d**) SEM micrograph of PVA/CHT/PHMB film removed from the surface of human skin.

Figure 3a clearly showed that the PVA/CHT/PHMB blends could easily form uniform and transparent films on the surface of human skin, and the film could be easily removed. Figure 3b,c showed digital and optical photograph of PVA/CHT/PHMB film removed from the surface of human skin. The skin wrinkle could be clearly seen on the film, which indicated that the film could adhere on the surface of human skin well. The SEM image of the PVA/CHT/PHMB blends formed film was presented in Figure 3d, and clearly showed plenty of 100 nm size around nano-phase in the film, which was correspondent to the phase separation in PVA/CHT/PHMB blends showed in Figure 1. The inherent nano-scaled phase enabled the film to obtain enhanced antibacterial ability. Hence, the above data indicated that the PVA/CHT/PHMB blends would be comfortably used in different parts of the body having kinds of size, shapes and contours, especially could be used in the joints including knees and elbows, and its transparent property could allow convenient inspection of the status of wound without removing it.

### 3.4. WVTR (Water Vapor Transmission Rate) of PVA/CHT/PHMB Blends Formed Film

**Figure 4:**
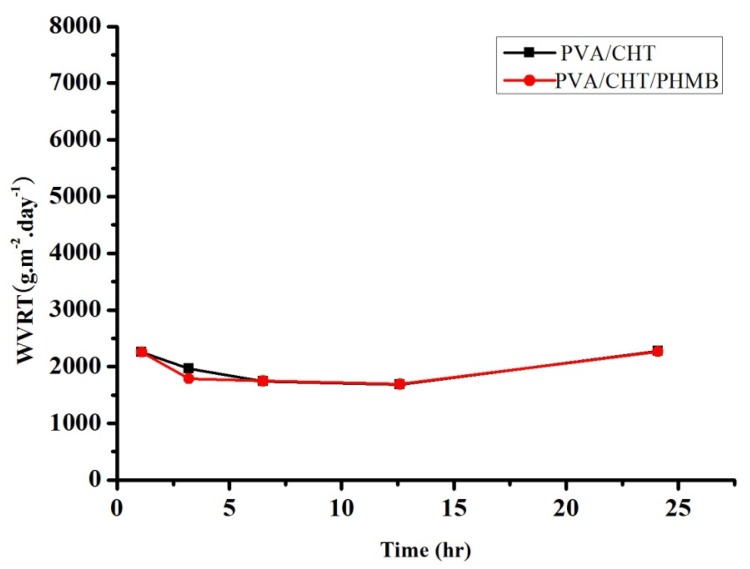
The WVTR of PVA/CHT/PHMB blends formed film.

WVTR is one of key parameters for wound dressings and proper WVTR is quite beneficial to wound healing [23]. The WVTR for the PVA/CHT/PHMB blends formed film was approximately 2000 g/m^2^/day (Figure 4), which indicated that the film could keep a moist environment around the wound and could support the cell growth and regeneration. The PVA/CHT/PHMB blends formed film had similar WVTR values as the wound dressings reported in the previous studies [23].

### 3.5. Mechanical Properties of PVA/CHT/PHMB Blends Formed Film

**Figure 5:**
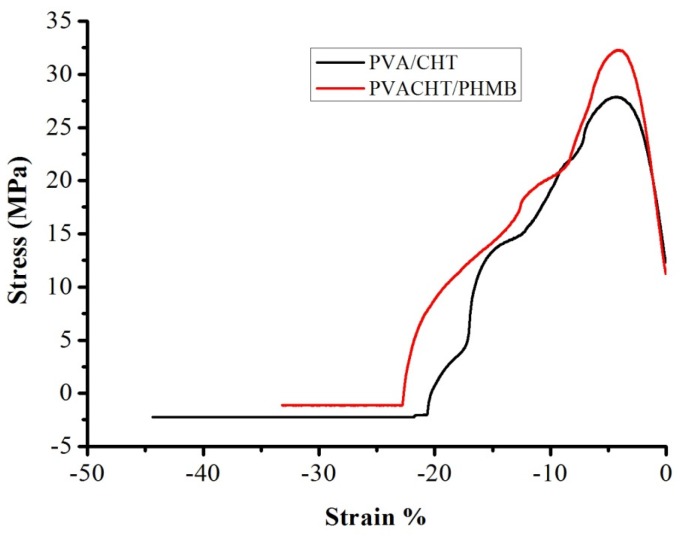
Strain versus strength of PVA/CHT/PHMB blends formed film.

The strain and stress were used to evaluate the mechanical properties of the PVA/CHT/PHMB blends formed film. We prepared PVA/CHT/PHMB blends formed film with length (10 mm), width (10 mm) and thickness (0.077 mm) to evaluate the mechanical property. The film exhibited good strength with 34 MPa around, and the strain of film was 4% around (Figure 5). Thus, the proper WVTR and mechanical property enabled the PVA/CHT/PHMB film to be possibly used as liquid bandage for wound healing, such as abrasion and tears caused by mechanical injuries.

### 3.6. Scratch Wound Healing Test

**Figure 6:**
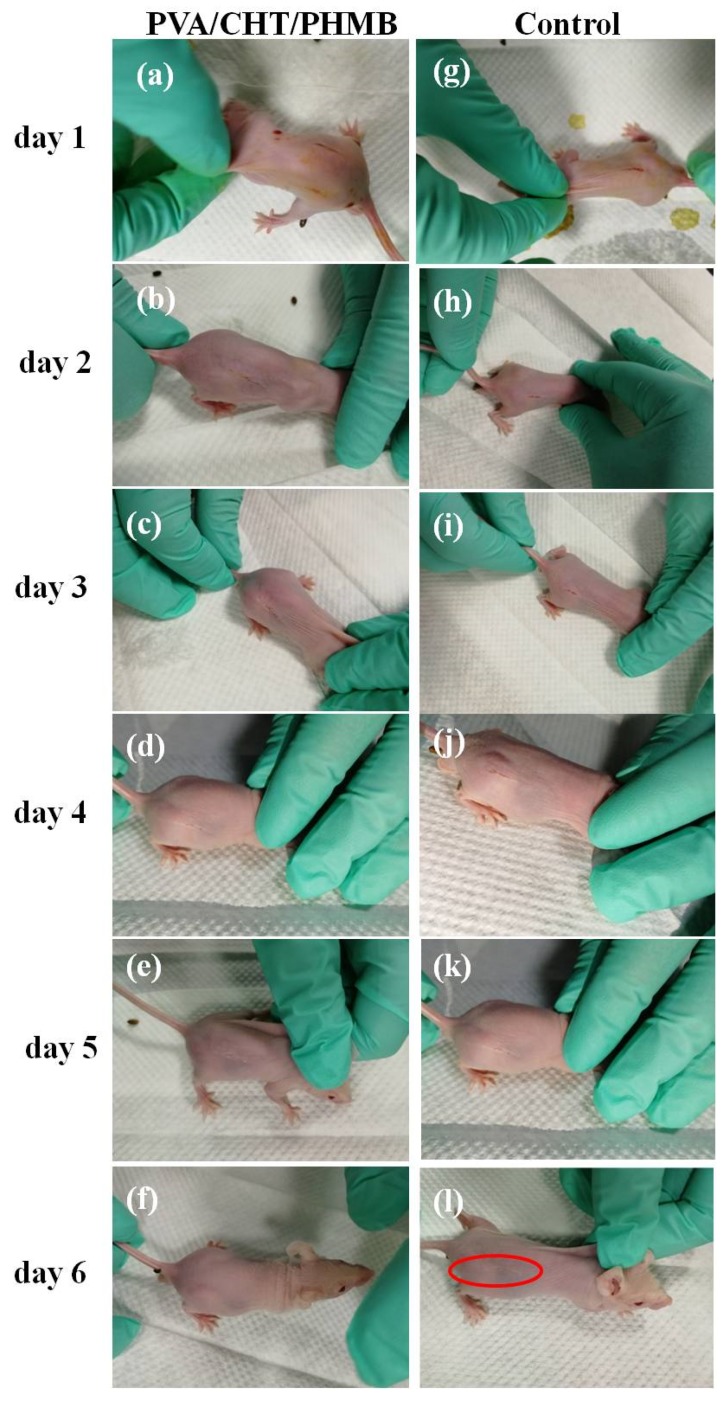
Scratch wound healing test in nude mouse. Compared with the control group (Figure 6**g**–**l**).

The nude mice were used to evaluate PVA/CHT/PHMB blends for the treatment of scratch wounds. The wound healing kinetics was characterized by wound appearance of wound closure, and the photographs of wound closure were provided in Figure 6. Compared with the control group (Figure 6g–l), the scratch wound was joining up better and was healed nicely after the wound was treated by PVA/CHT/PHMB blends for six days (Figure 6a–f). However, the scratch wound of the control group was not healed after six days. Therefore, the process of wound healing treated by PVA/CHT/PHMB blends was accelerated compared with control group, suggesting the PVA/CHT/PHMB blends could promote the scratch wound healing. In addition, due to the enhanced antimicrobial effect, water vapor permeability, flexible and transparent property of the blends, it was reasoned that the blends could potentially be used to treat localized infections, prevent infection following dermatologic surgery, abrasions, cosmetic procedures, and promote the wound healing.

## 4. Conclusions

In summary, a PVA/CHT/PHMB phase separation system has been successfully developed as a potential low dose PHMB-functionalized nano-topical antibacterial formulation with enhanced antimicrobial effect. The fabrication method was highly simple, just dissolved PVA, CHT, PHMB in water in order. There was the aggregates with size of 100 nm around induced by phase separation in the PVA/CHT/PHMB blends and an aqueous two-phase system formed, non-ionic polymer PVA formed continuous phase and cationic polymer CHT and PHMB formed dispersed phases. The MIC of PHMB in the blends was 0.5 μg/mL, which was four times lower than the MIC of PHMB individually. A phase separation increased zeta potential mechanism was proposed to explain the enhanced antibacterial activity of the blends. The blends could easily form film on the surface of skin with good water vapor transmittance rate and mechanical properties, and be used as liquid bandage to promote the scratch wound healing of the nude mouse. The PVA/CHT/PHMB blends could be potentially applied as a low dose topical antibacterial formulation with enhanced antimicrobial effect. Furthermore, the nano-sized phase separation strategy could be further explored as antibacterial system in medicine, especially in developing novel low dose topical antibacterial formulations.

## Figures and Tables

**Table 1:** The data of MIC assay.

MIC(µg/mL)	PHMB	PVA/PHMB	CHT/PHMB	PVA/CHT/PHMB
*S. aureus*	2	2	1	0.5
*E. coli*	2	1	1	0.5
